# Ferroptosis—A New Dawn in the Treatment of Organ Ischemia–Reperfusion Injury

**DOI:** 10.3390/cells11223653

**Published:** 2022-11-17

**Authors:** Linxiang Zhou, Shangting Han, Jiayu Guo, Tao Qiu, Jiangqiao Zhou, Lei Shen

**Affiliations:** 1 Department of Gastroenterology, Renmin Hospital of Wuhan University, Wuhan University, Wuhan 430060, China; 2 Department of Organ Transplantation, Renmin Hospital of Wuhan University, Wuhan University, Wuhan 430060, China

**Keywords:** ischemia–reperfusion injury, treatment, mechanism, ferroptosis, lipid peroxidation

## Abstract

Ischemia–reperfusion (I/R) is a common pathological phenomenon that occurs in numerous organs and diseases. It generally results from secondary damage caused by the recovery of blood flow and reoxygenation, followed by ischemia of organ tissues, which is often accompanied by severe cellular damage and death. Currently, effective treatments for I/R injury (IRI) are limited. Ferroptosis, a new type of regulated cell death (RCD), is characterized by iron overload and iron-dependent lipid peroxidation. Mounting evidence has indicated a close relationship between ferroptosis and IRI. Ferroptosis plays a significantly detrimental role in the progression of IRI, and targeting ferroptosis may be a promising approach for treatment of IRI. Considering the substantial progress made in the study of ferroptosis in IRI, in this review, we summarize the pathological mechanisms and therapeutic targets of ferroptosis in IRI.

## 1. Introduction

Ischemia–reperfusion injury (IRI) is one of the primary causes of organ failure in patients and refers to the phenomenon of increased or even irreversible damage after the restoration of blood flow due to various causes of organ ischemia [1]. IRI inevitably occurs during surgical procedures, organ transplantation, traumatic shock, and thrombotic diseases [1,2]. The combination of ischemia and reperfusion of the blood flow mediates renal tissue injury. Ischemia is the first key event in IRI, spearheading the induction of cellular metabolic imbalance and an abrupt decrease in oxygen availability in tissues; the second major incident is the recovery of blood flow and reoxygenation in the ischemia-involved region, where excess free radicals trigger destructive inflammatory responses and induce an overproduction of reactive oxygen species (ROS), which leads to worsening tissue damage or secondary injury [1,3]. Research on the role and mechanisms of IRI has been conducted in various organs, with studies focusing mainly on the heart, brain, kidney, liver, intestine, and lungs. Despite substantial research on IRI in recent years, the fine-grained mechanisms associated with IRI are extremely complex and remain elusive, hindering the implementation of pharmaceutical strategies. Accordingly, it is crucial to learn from experience and explore novel therapeutic targets for IRIs.

A unique and iron-dependent form of RCD known as ferroptosis is characterized by disordered iron metabolism and lipid peroxide buildup [4]. Since ferroptosis was discovered over the past decade, tremendous advances in understanding ferroptosis have shed light on its regulatory mechanisms and relevant disease contexts. Mounting evidence indicates that ferroptosis is one of the key drivers of the onset and progression of ischemic organ injury, and some small molecules or drugs with ferroptosis-inhibiting properties have succeeded in reducing IRIs in different organs. This review provides an overview of current developments in ferroptosis pathogenesis, ferroptosis evidence in organ IRIs, and associated potential therapeutic targets and strategies.

## 2. Mechanisms Governing Ferroptosis

**Figure 1:**
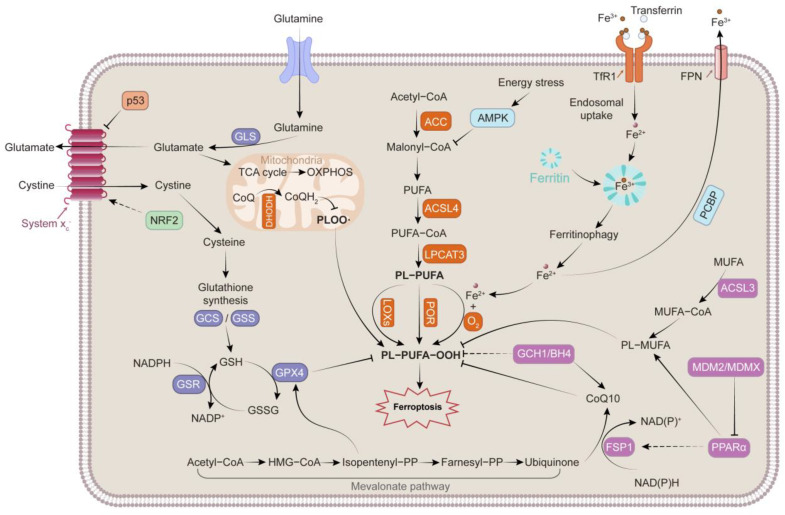
An overview of the mechanism and key regulators of ferroptosis. The mechanism of ferroptosis is complex and morphologically, genetically, and biochemically distinct from other forms of RCD. Iron metabolism, lipid peroxidation, and antioxidant system are involved in the mechanism of ferroptosis.

The process of ferroptosis differs from that of apoptosis, necrosis, and other types of RCD in terms of genetics, morphology, and biochemistry (Table 1) [4,5,6]. Multiple biological modulatory pathways, including disturbed iron homeostasis and lipid peroxidation, contribute to and collectively mediate ferroptosis. The mechanisms and main regulators involved in ferroptosis are presented in Figure 1.

### 2.1. Iron Metabolism

**Figure 2:**
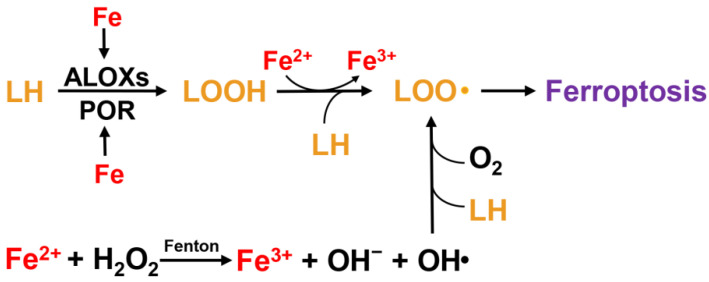
The effect of iron on lipid oxidation in ferroptosis.

At physiological concentrations, iron is an essential nutrient for the human body. Iron accepts electrons and donates them; therefore, abnormal accumulation of iron can lead to oxidative injury or even cell death. Iron in food exists mostly in the form of Fe^3+^, which must be reduced to Fe^2+^ or combined with iron chelators for absorption in the intestine. Cells acquire Fe^3+^ through transferrin (TF) and non-TF-bound iron (NTBI). In cases of Fe^3+^ overload, excess Fe^3+^ can exceed the burden capacity of TF and circulate as NTBI. Each TF can bind to two Fe^3+^ ions, and the TF carrying Fe^3+^ additionally binds to transferrin receptor 1 (TFR1), leading to membrane invagination and the formation of specialized endosomes [7]. In acidic endosomes, Fe^3+^ is released from TF and reduced to Fe^2+^ by six-transmembrane epithelial antigen of the prostate 3. Fe^2+^ traverses the endosomal membrane to the cytoplasm via divalent metal transporter 1 (DMT1), solute carrier family 39 member 14, and transient receptor potential mucolipin 1, constituting a dynamic labile iron pool (LIP) [8]. Additionally, heme metabolism mediated by heme oxygenase 1 (HO-1) can provide equimolar amounts of reactive Fe^2+^, carbon monoxide, and biliverdin Ixα [9,10], which also contributes to the accumulation of LIP. Mitochondria are the main organelles of iron influx in LIP, as the site of heme or iron-sulfur (Fe-S) clusters [11]. Mitochondrial iron homeostasis depends on several mitochondrial membrane proteins, including SLC25A37 and SLC25A28, which are the main importers of iron [12,13]. CDGSH iron sulfur domain 1 (CISD1) and CISD2 can restrict the uptake of mitochondrial iron [14]. Owing to the elevated Fe^2+^-driven Fenton reaction, hydroxyl radicals are produced by H_2_O_2_. By reacting with polyunsaturated lipids (LH), hydroxyl radicals generate lipid peroxyl radicals, and arachidonate lipoxygenases (ALOXs) can also oxidize LH into hydroperoxide (LOOH), which is then converted into lipid peroxyl radical by Fenton reaction. Eventually, the lipid peroxyl radicals initiate and facilitate lipid peroxidation [15,16]. Moreover, iron can enhance the activity of lipoxygenases (ALOXs) and cytochrome P450 oxidoreductase (POR), which are metabolic enzymes associated with phospholipid peroxidation (Figure 2). Iron is indispensable for the synthesis of several iron-containing enzymes associated with cellular ROS [17].

Intracellular iron is retained in ferritin as Fe^3+^, which attenuates the effects of Fe^2+^-mediated oxidative damage. Higher ferritin levels represent greater iron storage and resistance to ferroptosis. The degradation of ferritin by nuclear receptor coactivator 4 (NCOA4) and the ubiquitin proteasome system can lead to increased susceptibility to ferroptosis [18,19]. In mammalian cells, solute carrier family 40 member 1 (SLC40A1/FPN) facilitates intracellular iron export [20]. The current evidence indicates that overexpression of SLC40A1 improves ferroptosis [21]. Ferroptosis susceptibility is influenced by the dynamic modulation of iron between systemic and local cells.

### 2.2. Lipid Peroxidation

Unrestricted lipid peroxidation, which is the defining event of ferroptosis, is a process by which oxidants attack the carbon–carbon double bonds of lipids. Polyunsaturated fatty acids (PUFAs) are the main substrates of this process, especially arachidonic acid (AA) and adrenic acid (AdA) [22]. The weak C–H bonds at the bis-allylic positions of PUFAs make them susceptible to oxidation [23]. Moreover, PUFAs on the membrane are the primary targets of ROS [24]. Following the primary oxidation step, free radicals can migrate inside the same molecule or oxidize additional molecules [25,26]. By accelerating the addition of CoA to the long-chain polyunsaturated bonds of free AA/AdA and facilitating its esterification to phospholipids, acyl-CoA synthetase long-chain family member 4 (ACSL4) plays a critical role in ferroptosis. Subsequently, lysophosphatidylcholine acyltransferase 3 (LPCAT3) mediates the production of AA/AdA-CoA and membrane PE to generate AA/AdA-PE [8]. Thus, the inhibition or promotion of ACSL4 and LPCAT3 expression may be an effective means of desensitizing or sensitizing cells to ferroptosis. Finally, multiple oxidase enzymes (ALOXs, CYP/CYP450, prostaglandin–endoperoxide synthase 2 [PTGS2]/COX-2) directly oxidize lipids. ALOXs are non-heme, iron-dependent dioxygenases that function directly on PUFAs in biological membranes [17,27]. ALOXs mediate PUFA peroxidation to generate AA/AdA-PE-OOH, leading to the onset of ferroptosis, which can be promoted by phosphatidylethanolamine binding protein 1 through direct interaction with arachidonate 15-lipoxygenase (ALOX15) [28]. Studies have confirmed that ALOX knockout or the application of ALOX inhibitors can protect against organ damage by inhibiting ferroptosis [29,30,31]. Nevertheless, some ferroptosis-sensitive cell lines may not express ALOX enzymes [32], and in experimental models, knockout of 12/15-ALOX based on glutathione peroxidase 4^−/−^ (GPX4^−/−^) did not inhibit ferroptosis [33]. Despite these findings, the specific function of ALOXs in ferroptosis remains unclear. ALOXs are not the only enzymes that catalyze PUFA-PE. With nicotinamide adenine dinucleotide phosphate (NADPH) as the electron donor, POR can bind to flavin mononucleotide and flavin adenine dinucleotide and transfer electrons to downstream CYP/CYP450, driving lipid peroxidation of PUFAs [8,34]. PTGS2 could oxidize lysophospholipids and is considered an indicator of ferroptosis, although it is not a driver [35]. In addition to enzymatic peroxidation, a Fenton reaction is responsible for lipid peroxidation (as described for iron metabolism). Interestingly, increased synthesis and production of PUFAs upregulate sensitivity to ferroptosis; however, intracellular β-oxidation reduces the rate of lipid peroxidation by depleting most fatty acids [7]. The products of lipid peroxidation include early LOOH and subsequent increased toxic derivatives (MDA and 4-HNE) that can react with DNA, proteins, and other nucleophilic molecules [36], and extensive lipid peroxidation affects the integrity and permeability of cell membranes [37].

### 2.3. Antioxidant System

Depletion and inactivation of intracellular antioxidant defense systems facilitate lipid hydroperoxide accumulation. Most of the classical ferroptosis initiators (e.g., erastin and RSL3) also inhibit antioxidant systems [38]; the collapse of the antagonist system facilitates the outbreak of ferroptosis. GPX4 is a recognized gatekeeper of ferroptosis and functions centrally to prevent lipid peroxidation [7]. GPX4, a selenocysteine-containing phospholipid hydroperoxidase, could reduce peroxidized lipids, whether in free form or in combination with other lipids, such as PLs; proteins, such as lipoproteins; or within membranes [26,39,40]. The ping-pong mechanism is involved in the catalysis of lipid hydroperoxides by GPX4, whereby selenocysteine shifts between reduced selenol (SeH) and oxidized selenate (SeOH). First, SeOH is produced when SeH in GPX4 is oxidized by LOOH, whereas LOOH is reduced to nontoxic LOH. Upon reduction by GSH, SeOH becomes an intermediate selenide disulfide (Se-SG), which is subsequently reconverted to SeH by the second equivalent of GSH, releasing the byproduct GSSG, with NADPH acting as an electron donor and capable of being converted to GSH through glutathione reductase [7,41,42]. Hence, GSH plays a primary role in maintaining GPX4′s antioxidant capacity. The tripeptide, GSH, comprises cysteine, glycine, and glutamic acid. The availability of cysteine and glutamate-cysteine ligase within cells dictates GSH synthesis. Cystine is brought into the cell through system xc^−^, by which it is rapidly converted to cysteine for GSH synthesis [7]. As a crucial upstream gene of GPX4, intracellular solute carrier family 7 member 11 (SLC7A11) can be regulated by multiple genes and agents, which effectively modulates susceptibility to ferroptosis.

Coenzyme Q10 (CoQ10) is a ubiquitous intracellular compound that exerts electron-transfer functions and can be reduced to panthenol, which directly diminishes lipid radicals and terminates lipid autoxidation. Therefore, CoQ10 has been suggested as an essential endogenous inhibitor of ferroptosis. Owing to its NADH: ubiquinone oxidoreductase activity, ferroptosis suppressor protein 1 (FSP1) has been proven to reduce CoQ10 to generate panthenol, thus inhibiting lipid peroxidation in a GPX4-independent manner [17,43,44]. Current evidence indicates that tetrahydrobiopterin/dihydrobiopterin (BH4/BH2) synthesized by GTP cyclohydrolase 1 antagonizes ferroptosis; BH4 is involved in the synthesis of CoQ10 and can induce lipid remodeling by specifically preventing two polyunsaturated fatty acyl tails from the consumption of phospholipids [8,45,46].

In addition to GPX4 in the cytoplasm and mitochondria and FSP1 in the plasma membrane, dihydroorotate dehydrogenase (DHODH), an enzyme located on the outer side of the inner mitochondrial membrane, is a newly discovered cytoprotective system that antagonizes ferroptosis. The role of DHODH is similar to that of mitochondrial GPX4. In the mitochondrial inner membrane, DHODH reduces ubiquinone to ubiquinol, a free radical-trapping antioxidant that antagonizes phospholipid hydroperoxyl radical (PLOO•), thereby restraining ferroptosis. Mitochondrial GPX4 and DHODH are the two main mitochondrial defense systems that mitigate lipid peroxidation. Inactivation of DHODH (knockout or inhibitor treatment) in cancer cells with low GPX4 expression causes severe mitochondrial lipid peroxidation and ferroptosis. However, in cancer cells with high GPX4 levels, inactivation of DHODH exacerbates mitochondrial lipid peroxidation, in concert with ferroptosis inducers. In several GPX4 knockdown cell lines, restoration of GPX4mito but not GPX4cyto rescued the sensitivity of the cells to DHODH depletion [47].

It has been demonstrated that AMP-activated protein kinase (AMPK) exerts as a double-edged sword in ferroptosis. Acetyl-CoA carboxylase alpha is phosphorylated by AMPK, leading to impaired biosynthesis of PUFAs [48]; on the other hand, by inhibiting SLC7A11 and regulating autophagy, AMPK phosphorylates Beclin 1, resulting in ferroptosis [49]. Moreover, nearly all ferroptosis-related genes can be modulated by nuclear factor erythroid 2-related factor 2 (NRF2), which also plays a pivotal role in the antioxidant system by regulating intracellular iron homeostasis (e.g., FPN, FTH1, and FTL), redox regulation (e.g., GPX4, NQO1, and HO-1), GSH homeostasis (e.g., SLC7A11, GCLC, and GCLM), and NADPH generation (e.g., G6PD, PHGDH, and ME1) [26,50,51,52].

## 3. Mechanisms and Targeted Therapies for Ferroptosis in IRI

### 3.1. Ferroptosis and Myocardial IRI

Ischemic heart disease is the primary contributor to human death worldwide [53], and acute myocardial infarction caused by IRI is the primary cause of disability and death [54,55]. The occurrence of ischemia reduces the oxygen supply and contributes to the initial damage to the cardiac tissue [56]. Therefore, reoxygenation (primary percutaneous coronary intervention) is the preferred treatment [57]. Unfortunately, the reperfusion procedure inevitably induces cardiomyocyte death and increases infarct size, worsening the condition [53,58]. Among all types of organ IRI, ferroptosis in myocardial IRI has been the most extensively investigated. Ferroptosis primarily occurs during the period of myocardial reperfusion rather than ischemia [59]. As the damage caused by ischemia increased progressively, no significant differences were observed in the ferroptosis indices (GPX4, ACSL4, Fe^2+^, and MDA) in the heart tissue. Conversely, with prolongation of the reperfusion phase, a gradual increase in the ACSL4, Fe^2+^, and MDA levels, along with a decrease in GPX4 levels, was observed. Deferoxamine treatment attenuated myocardial injury and inhibited ferroptosis in I/R-treated rat hearts; however, no significant alleviating effect of deferoxamine was observed in the rat hearts that received only ischemic treatment. During ischemia, specific redox reactions of PUFA-phospholipids in cardiomyocytes are induced, which act as initiating signals to trigger intensive oxidative damage during the reperfusion phase. ALOX15, a critical enzyme for the oxidation of PUFA-phospholipids, is induced by ischemia/hypoxia [60]. The induction of ALOX15 functions as a “burning point” and initiates the oxidation of PUFA-phospholipids, particularly PUFA-PE, resulting in ferroptosis in myocardial cells. ALOX15 knockout mice lose PUFA-induced susceptibility to ischemia-induced cardiac injury. Liu et al. reported that activating transcription factor 3 (ATF3), expressed at the peak level in early reperfusion, and knockout of ATF3 remarkably aggravates IRI. In cardiomyocytes, ATF3 overexpression inhibits ferroptosis caused by erastin and RSL3. FA complementation group D2 promoter activity can be enhanced by binding of ATF3 to the transcriptional start site, providing evident antiferroptosis and cardioprotective effects against H/R injury [61].

Iron is stored in ferritin under physiological conditions. When intracellular iron deficiency occurs, ferritin-containing ions combine with NCOA4, which mediates ferritinophagy to release iron ions. DNMT-1 inhibition attenuates ferroptosis in diabetic myocardial I/R by regulating NCOA4-mediated ferritinophagy [62]. FPN1 is a unique protein that regulates iron release in mammals, whereas NRF2 regulates iron transcription. Upon release from the cytoplasm, NRF2 translocates into the nucleus and interacts with ARE promoter regions and sMaf proteins, leading to the upregulation of downstream protective proteins, such as FPN1 and GPX4, and a reduction in myocardial Fe^2+^ and MDA content [63]. Machado et al. demonstrated that upon cardiac IRI, ferritin heavy-chain (FTH) deficiency in the myocardium induces compensatory upregulation of several antiferroptotic proteins, including HO-1. Such upregulation of HO-1 results in the induction of SLC7A11 and supplementation of intracellular glutathione to inhibit the ferroptosis pathway, thereby preserving the function of the mitochondria and myocardium [64]. However, recent studies have revealed the negative effects of HO-1, making it a double-edged sword in ferroptosis. In response to H/R, HO-1 is upregulated and anchored in the endoplasmic reticulum (ER), leading to heme degradation and the production of Fe^2+^ with biliverdin and carbon monoxide, which induces the accumulation of Fe^2+^ in the ER, as well as the occurrence of ferroptosis. Silencing HO-1 eliminates iron overload in the ER [65].

Mitochondrial bioenergetics and GSH play major roles in ferroptotic cell death. An early response of cardiac mitochondria to ferroptotic stimuli may be implicated in the accumulation of ferroptotic phospholipid signals (especially PEox) in the mitochondria. Consequently, preservation of glutathione pools in the mitochondria is critical for the prevention of ferroptosis in cardiomyocytes [66]. Oxidized phosphatidylcholines (OxPCs) induce cardiomyocyte death in a concentration-dependent manner during I/R. Mechanistically, OxPCs can lead to a reduction in mitochondrial functional capacity, resulting in the disruption of calcium handling and contractile dysfunction, which can be attenuated by ferrostatin-1 and liproxstatin [67]. During myocardial I/R, forkhead box C1 binds to the ELAV-like RNA-binding protein 1 (ELAVL1) promoter region to initiate the transcription of ELAVL1, which can directly bind and stabilize Beclin 1 mRNA and drive ferroptosis by regulating autophagy, leading to overproduction of lipid peroxidation.

Ubiquitination reportedly regulates the degradation of hub genes in the ferroptosis pathway. According to a recent study, inhibition or knockdown of ubiquitin-specific peptidase 7 diminished H/R damage (reducing LDH release and necrosis), improved ubiquitination of p53, decreased p53 and TFR1 levels, and attenuated ferroptosis [68]. Ubiquitin-specific peptidase 22 stabilizes sirtuin 1 (SIRT1) through deubiquitination, and increased SIRT1 expression results in decreased p53 acetylation and protein expression. p53 binds to the SLC7A11 promoter to inhibit SLC7A11 expression. Therefore, p53 depletion increased GSH levels, but reduced ROS levels and lipid peroxidation [69].

The function of non-coding RNAs in ferroptosis has also been reported. Sun et al. reported that silencing lncAABR07025387.1 ameliorated myocardial IRI in vivo and restrained ferroptosis in cardiomyocytes during H/R. By interacting with miR-205, lncAABR07025387.1 upregulated ACSL4, a recognized promoter of ferroptosis [70]. A positive correlation was identified between miR-135b-3p levels and ferroptosis severity in myocardial I/R models. miR-135b-3p inhibits translation by binding to the 3′UTR of human GPX4 mRNA and promotes cellular ferroptosis, resulting in the exacerbation of myocardial IRI [71]. In murine cardiomyocytes, Mir9-3hg, an exosomal long non-coding RNA derived from bone marrow stem cells, inhibited I/R-induced ferroptosis by directly targeting the Pumilio 2 (PUM2)/PRDX6 axis. Incubation with BMSC-Exos enhanced GSH levels and diminished Fe^2+^ concentration, ROS, and ferroptosis biomarker expression in H/R-treated cells, whereas these effects can be reversed by intervention with Mir9-3hg [72].

Targeting ferroptosis is a promising strategy to alleviate and cure myocardial IRI. According to research by Li et al., ferrostatin-1 (Fer-1) prevented neutrophil recruitment after heart transplantation, decreased levels of 4-HNE, and reduced myocardial cell death. The effects of Fer-1 include improvement of left ventricular systolic function, a decrease in the size of myocardial infarctions, and left ventricular remodeling [73]. Cardiac remodeling and fibrosis were remarkably reduced in mice receiving an injection of Fer-1 or dexrazoxane every 2 days. Additionally, the reduction in mt-Atp6 and mt-Cytb mRNA levels in the heart caused by I/R was also prevented, indicating that ferroptosis inhibition substantially protects against myocardial I/R, possibly by preserving mitochondrial function. Additionally, liproxstatin-1 (Lip-1) provides cardiac protection by reducing the extent of myocardial infarct and maintaining mitochondrial integrity. Cardioprotection mediated by Lip-1 involves a reduction in voltage-dependent anion channel 1 (VDAC1) levels and oligomerization but not VDAC2/3. Furthermore, lip-1 administration rescued I/R-induced GPX4 depletion and limited the production of ROS in mitochondria [74].

Targeting NRF2 is an effective strategy against ferroptosis. Histochrome (HC) is used in clinical practice, owing to its potent antioxidant content and iron-chelating ability. The hearts of rats administered early intravenous injections of HC before reperfusion exhibited remarkably reduced cardiac fibrosis and increased capillary density. By inducing the expression of NRF2 and antioxidant genes, HC can reduce cytoplasmic and mitochondrial ROS, maintain intracellular GSH levels, and enhance GPX4 activity [75]. Wang et al. demonstrated that myocardial infarction was substantially reduced by dexmedetomidine (Dex), and heart function improved, along with diminished lipid peroxidation and Fe^2+^ accumulation. Studies have confirmed that Dex activates NRF2 through the AMPK/GSK-3β pathway to protect the heart from I/R-induced ferroptosis [76]. Similarly, britanin upregulates GPX4 expression via this pathway, and the knockdown of NRF2 blocks the protective effects of britanin against H/R-induced injury in H9C2 cells [77]. Gossypol acetic acid (GAA) exerts cytoprotective effects by inhibiting ferroptosis during I/R. GAA remarkably reduces myocardial infarct size, decreases lipid peroxidation, activates NRF2, and downregulates levels of PTGS2 and ACSL4 in both mRNA and protein [78]. Additionally, Lv et al. reported that etomidate activates NRF2/HO-1 by facilitating nuclear translocation of NRF2 to suppress I/R-induced ferroptosis and attenuate heart failure, pathological injury, myocardial fibrosis, and inflammation [79]. Naringenin (NAR) attenuated histopathological injury, inflammation, and lipid peroxidation in heart tissue treated with I/R by regulating NRF2. Erastin inhibits the ability of NAR to protect H9C2 cardiomyocytes exposed to H/R [80]. The antioxidant effect of xanthohumol can reduce the generation of lipid peroxide and ROS, chelate Fe^2+^, and regulate NRF2 and GPX4 protein levels in cardiomyocytes during Fe-SP and RSL3-induced ferroptosis [81].

In a study by Liu et al., ferulic acid facilitated energy production and decreased the AMP/ATP ratio by upregulating AMPKα2 expression, as well as inhibiting ferroptosis by enhancing the activity of antioxidant enzymes (SOD, GSH-Px, and CAT), which was similar to the effect of ferroptosis inhibitor Fer-1 [82]. Moreover, inhibition of glutamine catabolism, an important contributor to ferroptosis, reportedly reduces I/R-induced cardiac injury; for example, glutaminase inhibitor compound 968 can substantially inhibit lactate dehydrogenase release and ferroptosis during reperfusion [83]. Resveratrol (Res), a polyphenol with multiple bioactivities, was demonstrated to diminish oxidative stress and Fe^2+^ levels in I/R models and regulate USP19-Beclin 1 autophagy to inhibit ferroptosis [84]. An agent capable of protecting the myocardium from I/R damage is cyanidin-3-glucoside (C3G). C3G treatment can relieve oxidative stress, downregulate LC3II/LC3I and TFR1 levels, and upregulate FTH1 and GPX4 expression in oxygen–glucose deprivation/reoxygenation (OGD/R)-treated H9c2 cells. In addition to inhibiting USP19 and LC3II protein levels, C3G enhances the K11-linked ubiquitination of Beclin 1 [85]. A previous study demonstrated that propofol pretreatment in vitro and in vivo potently prevented I/R-induced myocardial injury [86]. p53 is known to be degraded by binding to murine double minute 2 (MDM2) [87], whereas this process can be facilitated by the phosphorylation of AKT at ser166 or 188 [88], and propofol inhibits ferroptosis through AKT phosphorylation [89]. In addition, with increasing focus being placed on the utilization of circadian medicine against IRIs, recent studies have demonstrated that targeting clock genes also limits ferroptosis. SR9009, an inhibitor of Rev-ERBs, exogenously alleviates myocardial IRI by suppressing ferritinophagy/ferroptosis signaling during type 2 diabetes mellitus onset, with significant reductions in ACSL4, NCOA4, and LC3B protein levels and evident changes in ferroptosis-related proteins, including ALOX-15 and DMT1 [90].

### 3.2. Ferroptosis and Renal IRI

The maintenance of renal function depends on adequate bilateral renal blood perfusion, with the kidneys receiving up to one-quarter of the cardiac output. Thus, the occurrence of renal IRI may be induced by any cause of systemic circulatory failure or partial deficiency of blood flow in the intrarenal circulation, and renal IRI always occurs during kidney transplantation, as well as cardiac and urological procedures [91]. Angeli et al. elucidated the critical function of the glutathione/GPX4 axis in preventing lipid oxidation in the kidneys and presented genetic proof that the knockout of GPX4 led to cell death in a pathologically relevant form of ferroptosis [92]. Evidence has confirmed that a spiroquinoxalinamine derivative named liproxstatin-1 inhibits ferroptosis in proximal tubule cells and in GPX4-knockout mice. By inducing hemoglobin deposition and worsening iron toxicity in the proximal tubules, hemopexin, a heme-scavenging protein, accumulates in the kidneys and aggravates acute kidney damage (AKI). This deleterious effect of hemoglobin and hemagglutinin in proximal tubule cells is restrained by deferoxamine and ferrostatin-1 [93]. Pannexin 1 (Panx1), a member of the ATP-releasing pathway family, is implicated in renal IRI by mediating ferroptosis. Panx1 deficiency enhances the expression of the cytoprotective chaperone protein HO-1 and suppresses ferritinophagy by regulating the MAPK/ERK pathway [94].

In a study by Sun et al., lncRNA TUG1 carried by USC-Exos modulated ASCL4-mediated ferroptosis by interacting with serine- and arginine-rich splicing factor 1 (SRSF1), thereby mitigating I/R-induced AKI [95]. MiR-182-5p and miR-378a-3p interfered with the expression of GPX4 and SLC7A11 by specifically interacting with their 3′UTR regions of mRNA, and renal damage was substantially reduced when miR-182-5p and miR-378a-3p were silenced [96].

Physiologically, legumain (Lgmn) is abundantly expressed in the proximal tubular cells as an asparaginyl endopeptidase. Lgmn deficiency alleviates acute tubular damage and inflammation brought on by I/R. In LgmnKO mRTECs, ferroptosis induced by hypoxia or erastin is reduced. Chaperone-mediated autophagy of GPX4 is inhibited by Lgmn deficiency [97]. The administration of an miR-3587 inhibitor remarkably increased the expression of HO-1 protein in H/R-treated renal tubular epithelial cells, along with increased GPX4 protein levels, enhanced cell viability, decreased MDA levels, reduced Fe^2+^ levels, and restored normal mitochondrial membrane potential [98]. Tryptophan degradation is mediated by kynurenine, with indoleamine 2,3-dioxygenase 1 (IDO) acting as a rate-limiting agent in this process. In RPTECs, IDO was upregulated by both hypoxia and reoxygenation, which, in turn, triggered GCN2K-mediated apoptosis and AhR-mediated ferroptosis. IDO elevation occurs during both ischemia and reperfusion stages; therefore, inhibition of IDO can be an effective therapeutic strategy to prevent or mitigate IRI [99]. Inositol-requiring enzyme 1 (IRE1), a proximal ER stress sensor, activates the c-Jun N-terminal kinase (JNK) pathway in response to ER stress. Blood urea nitrogen, creatinine, and tissue damage in renal I/R-treated mice were significantly reduced by inhibition of IRE1/JNK, and the biomarkers of ferroptosis, such as 4-HNE and GPX4, were altered, as well as in H/R-induced IRE1/JNK knockdown HK-2 cell lines [100].

Evidence indicates that a positive relationship exists between renal IRI and lysine-specific demethylase 1 (LSD1) and that inhibition of LSD1 can alleviate I/R-induced damage in vitro and in vivo. LSD1 exacerbated ferroptosis and oxidative stress in the kidneys by decreasing H3K9me2 enrichment in the toll-like receptor 4 (TLR4) promoter region to activate the TLR4/NOX4 pathway [101]. ELAVL1 has been suggested as a crucial regulator of ferritinophagy that promotes ferroptosis. Under H/R, hydroxychloroquine (an autophagy inhibitor) or si-ELAVL1 reverses CIRBP-enhanced ferritinophagy activation and ferroptosis in HK-2 cells. Ferroptosis is inhibited by anti-CIRBP antibody injection in mice, and renal IRI is attenuated [102].

Linkermann et al. synthesized a novel ferrostatin (16–86) capable of suppressing ferroptosis in vivo. Compared to Fer-1, 16–86 proved to be more stable and potent. Even in situations with extremely severe renal IRI, 16–86 can exert a strong protective effect, a level of protection that has never been attained [103]. Zhang et al. reported that treatment with irisin (250 μg/kg) attenuated renal injury, inhibited the inflammatory response, enhanced mitochondrial function, and decreased ER stress and oxidative stress during renal I/R. RSL3, a GPX4 inhibitor, abolished the protective action of irisin [104]. A mitochondrially targeted nitroxide with a dual antioxidant effect, XJB-5-131, has been proven to effectively rescue mitochondrial function. In mice, XJB-5-131 exhibits excellent plasma stability, rapid plasma–kidney transfer, and high renal affinity. XJB-5-131 therapy results in elevated GSH-Px levels and reduced production of renal MDA, and 4-HNE staining also results in reduced lipid peroxidation [105]. Apart from cardiac I/R, Dex exerted protective effects against renal IRI. Dex administration attenuated renal tissue damage, restrained ferroptosis, and suppressed the inflammatory response, which is related to the inhibition of ACSL4. Furthermore, the protection provided by Dex for HEK293T cells against ferroptosis and inflammation brought on by OGD/R was abrogated by ACSL4 overexpression [106]. An inhibitory effect of quercetin (QCT) on ferroptosis was identified to be achieved via ATF3 inhibition. ATF3 knockdown remarkably elevated SLC7A11 and GPX4 levels and increased cell survival. Ferroptotic cells recruited macrophages via the C–C motif chemokine ligand 2, whereas QCT inhibited macrophage chemotaxis induced by ferroptosis in AKI [107]. The protective role of pachymic acid in I/R-induced AKI in mice can likely attributed to the activation of NRF2 and downstream (GPX4, SLC7A11, and HO-1) [108]. The antioxidant melatonin, which regulates the sleep–wake cycle, influences oxidative stress by alleviating the H/R-mediated reduction in NRF2 and increase in SLC7A11 in mouse tubular epithelial cells. Specific knockdown of NRF2 enhances cellular susceptibility to ferroptosis, and melatonin is unable to prevent ferroptosis under H/R conditions [109]. In a study by Yang et al., entacapone increased p62 expression and affected the p62–KEAP1–NRF2 pathway, thereby upregulating the nuclear translocation of NRF2. This function leads to upregulation of downstream SLC7A11 and substantial inhibition of oxidative stress and ferroptosis [110].

### 3.3. Ferroptosis and Cerebral IRI

Ischemic stroke is a severe public health issue, with high morbidity, disability, and fatality rates. Focal cerebral ischemia and hypoxia due to interruption of cerebral blood flow are the main pathological mechanisms leading to ischemic stroke [111], with ferroptosis being a major contributor to the pathogenesis of cerebral IRI and neuronal death. Iron accumulation and redistribution, glutamate accumulation, oxidative stress, lipid peroxidation, and epigenetic regulation are involved in the progression of ferroptosis in ischemic stroke [112].

Ferritin is a key component of iron homeostasis; Chen et al. observed ferritin to be significantly downregulated in a rat middle cerebral artery occlusion model, which, in turn, promoted p53 expression to inhibit SLC7A11 to induce ferroptosis in hippocampal neurons. Ferritin also significantly reduces tau hyperphosphorylation and oxidative stress [113]. Accordingly, the role of the Tau protein is age-dependent in cerebral IRI [114], and Tau can promote iron transfer in aging-related ischemic stroke to mitigate ferroptosis [115]. Additionally, NCOA4 mediates the excessive degradation of ferritin caused by ferritinophagy during cerebral I/R followed by ferroptosis [116].

Normally, restoring blood flow to the brain within an effective therapeutic time window is an important and unique approved treatment for ischemic stroke, as reperfusion can lead to secondary brain damage, and reducing ischemic reperfusion injury is another promising approach for ischemic stroke therapy. GPX4 may act as a critical regulator of ferroptosis, and retinoid X receptor γ can bind to the GPX4 promoter to activate its transcription and reduce IRI in the brain [117]. SLC7A11 mediates the synthesis of glutathione, which is available to GPX4 to reduce lipid peroxidation. However, PUM2 attenuates the inhibitory effect of SLC7A11 on ferroptosis by suppressing SIRT1 expression, thus exacerbating I/R-induced neuroinflammation and brain injury [114]. In tert-butyl-hydroxyperoxide-treated neurons, activation of the SSAT1/ALOX15 axis downregulated the expression of GPX4 and SLC7A11, which triggered neuronal death and worsened IRI [118]. Neural stem cell transplantation treated with neuregulin1β can regulate ferroptosis in I/R by promoting GPX4/SLC7A11 expression [119].

Several drugs have demonstrated efficacy against brain I/R, and some of these agents target ferroptosis. Baicalein inhibits ferroptosis in cerebral IRI by increasing GPX4 and ACSL3 expression, decreasing ACSL4 levels, and reducing Fe^2+^ accumulation and lipid peroxidation [120]. NRF2 is also a key regulator of ferroptosis. Rehmannioside A remarkably increased SLC7A11 and GPX4 protein levels in H_2_O_2_-induced neuronal injury by promoting the PI3K/AKT/NRF2 pathway, attenuating ferroptosis, and improving cell survival [121]. β-Caryophyllene also remarkably enhances the nuclear translocation of NRF2 and promotes HO-1 and GPX4 expression to inhibit ferroptosis in cerebral IRI [122]. Kaempferol can also alleviate I/R-induced neuronal ferroptosis by promoting GPX4 and SLC7A11 expression in an NRF2-dependent manner [123]. Galangin can inhibit ferroptosis in hippocampal neurons by directly upregulating SLC7A11 and thus indirectly upregulating GPX4, thereby reducing cerebral IRI [124]. Carvacrol also induces GPX4 expression and enhances the resistance to ischemic damage in hippocampal neurons [125]. Carthamin yellow protects against cerebral IRI in rats by reducing inflammation and interfering with ferroptosis [126]. Additionally, resveratrol inhibits neuronal ferroptosis induced by RSL3 or I/R treatment in rat brains and promotes neuronal survival [127].

Lipid peroxidation and ferroptosis are closely linked to mitochondrial and Golgi dysfunctions. The neuroprotective agent UBIAD1, a recently identified antioxidant enzyme, catalyzes the generation of CoQ10 in Golgi membranes, which regulates I/R-mediated ferroptosis by repairing mitochondrial and Golgi functions in damaged brain tissue and neurons [128]. Mainly expressed in cells with high oxygen demand, mitochondrial ferritin is a protein that stores Fe^3+^, which can limit iron overload and iron-dependent lipid peroxidation induced by cerebral IRI [129]. Mitochondrial ferritin overexpression attenuates ferroptosis in brain endothelial cells, which prevents disruption of the blood–brain barrier [130].

A multi-omics analysis identified thrombin and ACSL4 as critical proteins involved in ferroptosis in ischemic stroke. The product of thrombin metabolism, arachidonic acid, is esterified by ACSL4 and contains phosphatidylethanolamine arachidonic acid, which is a major product of iron-induced peroxidation and is involved in neuronal ferroptosis [131]. Prevention of ferroptosis in brain I/R leads to deactivation of COX-2/prostaglandin E2 (PGE2) signaling and reduces PGE2 release to attenuate brain IRI; in turn, PGE2 can inhibit brain I/R-induced ferroptosis by effectively reducing Fe^2+^, glutathione oxidation, and lipid peroxidation [132]. This depends largely on the different receptors that prostaglandin E2 influence. Furthermore, lncRNA PVT1 regulates ferroptosis in cerebral I/R via miR-214-mediated p53 and TFR1 [133]. These findings strongly support the hypothesis that ferroptosis is intimately implicated in the pathology of cerebral IRI and likely modulates its severity.

### 3.4. Ferroptosis and Intestinal IRI

Intestinal IRI is a clinical intestinal disease with high morbidity and fatality rates. Hemorrhagic shock, traumatic shock, strangulated intestinal obstruction, severe burns, and chronic and acute mesenteric ischemia are some of the pathologies that can lead to its incidence [134,135]. The main pathophysiological mechanism is mechanical occlusion of the blood flow in the intestinal vessels, which may also be secondary to severe interruption of blood flow to other internal organs, as well as tissue changes caused by arterial ischemia and hypoxia, which, in turn lead, to cellular damage, which may be further aggravated when the blood supply is suddenly restored [136,137,138]. Recent evidence has indicated that ferroptosis contributes to the pathogenesis described above.

ROS production and lipid peroxidation are closely associated with intestinal IRI, both of which are primary factors that initiate and execute ferroptosis. GSH levels and superoxide dismutase activity in rat intestinal tissues decreased, and MDA levels increased after intestinal I/R. Dioscin delivery is beneficial in reducing oxidative stress and lipid peroxidation by modulating miR-351-5/Sirt6 and is a possible therapeutic candidate for the mitigation of intestinal IRI [139,140]. Knockdown of NRF2 dramatically reduced SLC7A11 and HO-1 levels, NRF2/HO-1 expression in the OGD/R model was elevated, owing to the regulation of SLC7A11, and cell death was substantially reduced, confirming that NRF2 can suppress ferroptosis by modulating SLC7A11 and HO-1 [141]. Previous studies have revealed that the inhibition or knockdown of HO-1 and MAO-B reduces endothelial cell loss and protects the vascular endothelium after reperfusion. Apigenin-7-O-β-D-(-6″-p-coumaroyl)-glucopyranoside (APG), a flavonoid glycoside with strong antioxidant capacity, specifically binds to HO-1 and MAO-B. APG also attenuates ROS generation and Fe^2+^ accumulation, thus inhibiting ferroptosis in intestinal IRI in a dose-dependent manner [142].

Capsiate (CAT) is a metabolite of intestinal flora, and preoperative fecal CAT levels in patients with extracorporeal circulation are negatively correlated with intestinal IRI. The alleviation of CAT in intestinal IRI can be abrogated by RSL3. By activating transient receptor potential cation channel subfamily V member 1, CAT induces upregulation of GPX4 expression and prevention of ferroptosis [143]. The inhibition of ACSL4 prior to reperfusion has been demonstrated to be effective in preventing ferroptosis and cell death. Special protein 1 (Sp1) was identified as an essential contributor to ACSL4 expression in intestinal IRI. As a key transcription factor that promotes ACSL4 expression, Sp1 enhances ACSL4 transcription by binding to its promoter region of ACSL4 [144]. Ferroptosis has also been reported to be involved in intestinal I/R-induced acute lung injury (ALI). iASPP reportedly inhibits ferroptosis, in part via NRF2 signaling, thereby attenuating intestinal I/R-induced ALI. The protective function of iASPP against intestinal I/R-induced ALI is mediated through stimulation of NRF2/HIF-1/TF [145].

### 3.5. Ferroptosis and Hepatic IRI

Hepatic IRI can be induced by shock (sepsis and bleeding) or liver surgery (liver transplantation or partial hepatectomy). Severe hepatic IRI can result in hepatic impairment and acute liver failure [146,147]. IRI occurs as a result of oxidative stress, along with nutritional deficiency, diminished circulation supply, and inflammation [148]. Iron-mediated cell death may be related to oxidative stress caused by ROS, particularly during blood reperfusion [149].

Ferroptosis caused by GPX4 inactivation is recognized as a contributing factor to hepatic IRI, and lip-1 can effectively diminish hepatic injury and substantially ameliorate hepatic function [92]. Yamada et al. studied the role of ferroptosis in hepatic IRI by establishing mouse models. Hepatic I/R significantly induced upregulation of PTGS2 (a ferroptosis marker), lipid peroxidation, and hepatic injury. Additionally, all hepatocyte injuries were reduced by Fer-1 and iron chelation. Thus, iron overload may be a critical factor in hepatic IRI [147]. In a retrospective study of 202 pediatric living donors for liver transplantation (LT), iron overload in the donor was identified as an independent risk factor for hepatic injury after LT, and ferroptosis was identified as a pathogenesis of hepatic IRI. Moreover, in a mouse model of hepatic IRI, hepatic injury, lipid peroxidation, inflammatory response, and the increase in PTGS2 were considerably attenuated by Fer-1 or α-tocopherol. Iron chelation effectively attenuates hepatic IRI, whereas iron overload induced by a high-iron diet aggravates hepatic IRI [147].

Previous studies have demonstrated that both inflammatory cytokines and cellular markers can be inhibited by Fer-1. Ferroptosis during hepatocyte death may be closely related to the inflammatory response in hepatic IRI. It has been hypothesized that the inflammatory reaction brought on by hepatic IRI leads to iron accumulation in hepatocytes. Increasing evidence suggests that iron plays a central role in numerous aspects of the innate immune response, including ROS generation and modulation of host inflammation. Iron overload leads to metabolic disorders, and defective nutritional immunity resulting from oversaturation of host transferrin increases susceptibility to infection [147,150,151].

Moreover, maresin conjugate in tissue regeneration 1 (MCTR1) ameliorates ferroptosis in hepatic IRI by promoting the nuclear aggregation of NRF2. MCTR1 treatment reduced iron content and serum LDH release levels, as well as ROS, MDA, IL-1β, COX2, and TFRC levels, in I/R-treated mice and OGD/R AML12 cells. Additionally, the levels of IL-10, GSH, and antioxidant biomarkers SOD and GPX4 were upregulated by MCTR1 [152]. miR-29a-3p in exosomes from HO-1-modified BMSC significantly ameliorated steatotic hepatic IRI in vivo and in vitro by suppressing ferroptosis via IREB2. The protective function of HO-1/BMMSC exosomes against IRI in SHP-HR cells was abrogated by downregulation of miR-29a-3p [153]. Macrophage extracellular traps (METs) have therapeutic potential to reduce hepatic IRI. Remarkable formation of hepatocyte METs occurs in patients undergoing hepatectomy with portal occlusion, as well as in mice with hepatic IRI. Interestingly, the activation of ferroptosis and decreased hepatocyte survival induced by I/R can be reversed by MET inhibition [154].

### 3.6. Ferroptosis and Pulmonary IRI

The lungs are fragile and susceptible to IRI. Pulmonary I/R raises the Fe^2+^ content and lipid peroxidation buildup, and key protein (GPX4 and ACSL4) levels change upon reperfusion. Lip-1 pretreatment diminishes ferroptosis and appreciably reduces lung injury caused by I/R in vitro and in vivo.

ACSL4, a crucial enzyme implicated in ferroptosis, has been confirmed to reduce pulmonary IRI. Xu et al. reported that ACSL4 knockdown blocked the accumulation of lipid peroxidation and decreased the susceptibility of lung epithelial cells to ferroptosis [155]. Additionally, administration of rosiglitazone (an ACSL4 inhibitor) prior to ischemia reduced ferroptotic damage of lung tissue upon reperfusion [155]. Signal transducer and activator of transcription 3 (STAT3) can be phosphorylated to pSTAT3 in response to the onset of tissue injury and serves as a warning sign to reinforce inflammation. Studies have confirmed that NRF2 is upregulated in OGD/R-treated MLE12 cells and drives STAT3 phosphorylation to amplify downstream signaling and cascade responses. Subsequently, the activation of pSTAT3 inhibits OGD/R-induced ferroptotic damage in MLE12 cells and pathological processes associated with ALI by modulating SLC7A11 [156]. Irisin, a muscle-derived myokine with potent protective effects, inhibits ferroptosis in pulmonary IRI both in vitro and in vivo [157]. Consistent with the action of Fer-1, inhibition of ferroptosis in pulmonary IRI by irisin was manifested by lower ROS, MDA, and Fe^2+^ levels and increased the expression levels of GPX4, which was achieved by promoting NRF2/HO-1 signaling. The cytoprotective capacity of irisin is abrogated when NRF2 is silenced [158]. Lidocaine inhibits ferroptosis in H/R-treated lung epithelial cells via the p38/MAPK pathway. Intracellular ROS and iron levels were diminished by lidocaine administration in a concentration-dependent manner. Moreover, lidocaine upregulated the expression levels of FTH1, GPX4, and SOD. Pretreatment with p79350 (an agonist of p38) partially weakens the protective function of lidocaine against H/R-treated lung epithelial cells [159].

**Figure 3:**
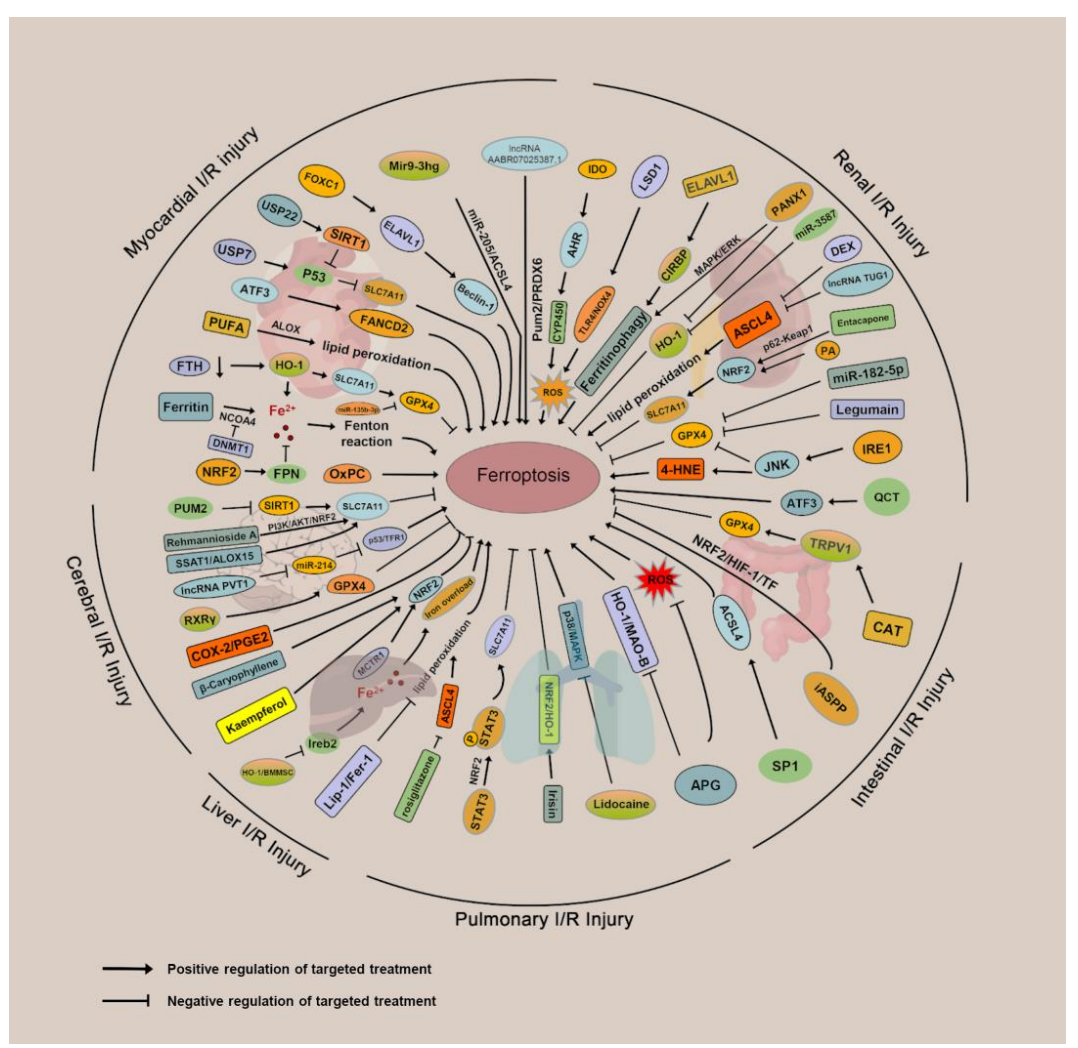
Pathological mechanisms of ferroptosis in organ ischemia–reperfusion injury diseases.

HO-1 expression levels are associated with increased tissue iron and ferritin levels, as well as inflammatory/antioxidant load. Human lung allografts suffering from acute cellular rejection and occlusive capillary bronchitis exhibit elevated HO-1 expression levels [160]. Furthermore, abnormally high levels of Fe and its homeostasis proteins were detected in lung allografts, with levels possibly increasing with time. The disruption of iron homeostasis after transplantation indicates that iron-depleting therapy is a potential strategy for prevention of lung allograft injury [160,161]. Similarly, in a study by Liu et al., untreated lung allografts were reported to contain higher concentrations of iron, which can be substantially diminished by pirfenidone, thereby limiting acute pulmonary allograft injury [162]. In Figure 3 and Table 2, we provide an overview of mechanisms and targeted therapies for ferroptosis in organ IRI.

## 4. Concluding Remarks and Future Perspectives

In the past few years, numerous studies have demonstrated that ferroptosis contributes to organ IRI, making it a promising research route. Disturbances in iron metabolism, oxidative stress, lipid peroxidation, and epigenetic regulation have been implicated in the progression of ferroptosis in I/R. In addition to apoptosis, ferroptosis plays an integral role in the mechanism of IRI, making the development of clinical agents targeting ferroptosis among the promising strategies for the early mitigation of IRI. Apart from exploring more selective and stable agents to specifically target ferroptosis, expansion of the toolset and markers is necessary for the accurate detection of ferroptosis. Research on ferroptosis has grown exponentially, and the role and mechanisms of ferroptosis in organ IRI have been continuously reported and updated. However, the specific physiological functions of ferroptosis remain ambiguous. In this paper, we summarize the recent relevant studies, providing clues for the clinical treatment of organ IRI.

Inhibition of ferroptosis, either by chemical agents (iron chelators, synthetic compounds, natural monomers, etc.) or genetic interventions, demonstrates limited yet encouraging success in the treatment of IRI. Nevertheless, colliding with this enthusiasm, many unresolved problems remain with respect to ferroptosis studies on IRI. IRI activates multiple types of cell death, especially apoptosis, which has been extensively investigated over the past 50 years. Although the inhibition of ferroptosis has been demonstrated to alleviate IRI, the weight and degree of importance of ferroptosis in regulated cell death remain unclear (in particular, compared to apoptosis), which, to some extent, determines the therapeutic efficiency of ferroptosis targeting. Ferroptosis in IRI may interact with other cell death modalities; however, the exact mechanism remains unknown, and a combination of multiple therapeutic approaches may lead to better outcomes. Importantly, most of the available studies on ferroptosis pathology have been conducted at the animal and cellular levels, and the application of these results to clinical settings requires further exploration. Iron metabolism and ROS are indispensable for the preservation of normal intracellular homeostasis and physiological functions; therefore, the appropriate therapeutic concentration intervals for iron chelators and anti-ROS agents in different organ IRIs still need to be explored to avoid side effects. Moreover, numerous targets for the activation and inhibition of ferroptosis in IRI have been revealed; however, the mechanism that dominates and provides the best therapeutic effect remains to be elucidated. In conclusion, research on ferroptosis in IRI is currently in its early stages, although targeting ferroptosis may be a promising therapeutic approach for IRI that deserves further research.

## Figures and Tables

**Table 1:** Comparison: features of different forms of RCD.

	Morphological Features	Biochemical Features	Core genes	Inducers	Inhibitors
Ferroptosis	Mitochondrial shrinkage and morphological abnormalities (increased membrane density, diminished or vanished cristae, ruptured outer membrane), with normal nucleus	Iron accumulation, lipid peroxidation, inhibition of SLC7A11/GSH/GPX4	GPX4, TFR1, SLC7A11, NRF2, NCOA4, P53, ALOXs, ACSL4, FSP1	Erastin, RSL3	Ferrostatin-1, liproxstatin-1, vitamin E, desferoxamine
Apoptosis	Cell shrinkage, plasma membrane blebbing, chromatin agglutination, nuclear fragmentation, apoptotic bodies	DNA fragmentation, activation of caspase pathway	Caspase, Bcl-2, Bax, P53, Fas	FASL, DCC, UNC5B	zVAD-FMK, XIAP, c-IAP1
Necroptosis	Swelling and rupture of cells and organelles, leakage of cell contents, moderate condensation of chromatin	Drop in ATP levels	RIP1, RIP3, LEF1	zVAD-fmk, TNF-α	Necrostatin-1, NSA
Pyroptosis	Cell swelling and plasma membrane bubbling, formation of inflammasome, release of cellular components	Activation of caspase-1 and GSDMD, release of pro-inflammatory cytokines	Caspase-1, IL-1β, IL-18, GSDMD	Lipopolysacc-haride, ivermectin	NAC, GSH
Autophagy	Formation of double-membraned autolysosomes	Increased lysosomal activity	ATG5, ATG7, LC3, BECN1, DRAM3, AMPK, mTOR	Rapamycin, valproate	Chloroquine, 3-methyladenine, wortmannin, Spautin-1, bafilomycin A1

**Table 2:** Potential therapeutic agents for organ I/R injury targeting ferroptosis.

I/R Injury Model	Reagents	Target	References
Myocardial I/R injury	Ferrostatin-1	Inhibit lipid peroxidation	[73]
	Deferoxamine	Chelation of iron	[59,163]
	Dexrazoxane	Chelation of iron	[164]
	Liproxstatin-1	Reduce ROS levels/VDAC1	[74]
	Histochrome	Increase expression of NRF2	[75]
	Dexmedetomidine	AMPK/GSK-3β/Nrf2	[76]
	Britanin	AMPK/GSK3β/Nrf2	[77]
	Gossypol acetic acid	Inhibit lipid peroxidation	[78]
	Etomidate	Nrf2/HO-1	[79]
	Naringenin	Nrf2/System xc-/Gpx4	[80]
	Xanthohumol	Inhibit lipid peroxidation /Chelation of iron	[81]
	Ferulic acid	Increase activity of antioxidant enzymes/AMPKα2	[82]
	Compound 968	Inhibit glutamine catabolism	[83]
	Resveratrol	Reduce oxidative stress/ USP19-Beclin1	[84]
	Cyanidin-3-Glucoside	Inhibit oxidative stress	[85]
	Propofol	microRNA-451/HMGB1	[86]
	SR9009	Inhibit ferritinophagy	[87]
Renal I/R injury	Liproxstatin-1	Inhibit lipid peroxidation	[92]
	Deferoxamine	Chelation of iron	[93]
	Ferrostatin-1	Inhibit lipid peroxidation	[103]
	16-86	Inhibit lipid peroxidation	[103]
	XJB-5-131	GPX4, ACSL4	[105]
	Dexmedetomidine	Inhibit ACSL4 via α2-AR	[106]
	Quercetin	ATF3	[107]
	Pachymic acid	NRF2/SLC7A11/GPX4	[108]
	Melatonin	NRF2/Slc7a11	[109]
	Entacapone	p62/KEAP1/NRF2/Slc7a11	[110]
Cerebral I/R injury	Baicalein	GPX4/ACSL4/ACSL3	[120]
	Rehmannioside A	PI3K/AKT/Nrf2 and SLC7A11/GPX4	[121]
	β-Caryophyllene	NRF2/HO-1	[122]
	Kaempferol	Nrf2/SLC7A11/GPX4	[123]
	Ferrostatin-1	Inhibit lipid peroxidation	[115]
	Galangin	SLC7A11/GPX4	[124]
	Carvacrol	GPX4	[125]
	Carthamin yellow	Fe^2+^/ROS/lipid peroxidation	[126]
	Resveratrol	Fe^2+^/ROS	[127]
	Liproxstatin-1	Inhibit lipid peroxidation	[115]
Hepatic I/R injury	Ferrostatin-1	Inhibit lipid peroxidation	[92,147]
	Deferoxamine	Reduce intracellular iron	[147]
	Liproxstatin-1	Inhibit lipid peroxidation	[115]
	α-tocopherol	Inhibit lipid peroxidation	[147]
Intestinal I/R injury	APG	HO-1 and MAO-B inhibition	[142]
	Dioscin	miR-351-5p/oxidative stress	[139]
	Liproxstatin-1	Inhibit lipid peroxidation	[144]
	Rosiglitazone	Inhibit ACSL4	[144]
	Capsiate	GPX4, TRPV1	[143]
Lung I/R injury	Liproxstatin-1	Inhibit lipid peroxidation	[155]
	Lidocaine	p38 MAPK pathway	[159]
	Rosiglitazone ACSL4	ACSL4	[155]
	Irisin	Nrf2/HO-1	[158]
	Pirfenidone	Reduce iron levels	[162]
	Ferrostatin-1	Inhibit lipid peroxidation	[158]

## Data Availability

Not applicable.

## Abbreviations

4-HNE	4-hydroxynonenal
AA	arachidonic acid
ACSL4	acyl-CoA synthetase long-chain family member 4
AdA	adrenic acid
AKI	acute kidney injury
ALI	acute lung injury
ALOX	arachidonate lipoxygenases
AMPK	AMP-activated protein kinase
APG	apigenin-7-O-β-D-(-6′′-p-coumaroyl)-glucopyranoside
ATF3	activating transcription factor 3
BH4/BH2	tetrahydrobiopterin/dihydrobiopterin
BMSC	bone marrow mesenchymal stem cells
C3G	cyanidin-3-glucoside
CAT	capsiate
CIRBP	cold inducible RNA binding protein
CISD1	CDGSH iron sulfur domain 1
CISD2	CDGSH iron sulfur domain 1
CoQ	coenzyme Q
CoQ10	coenzyme Q10
Dex	dexmedetomidine
DHODH	dihydroorotate dehydrogenase
DMT1	divalent metal transporter 1
ELAVL1	ELAV like RNA binding protein 1
ER	endoplasmic reticulum
Fer-1	ferrostatin-1
FSP1	ferroptosis suppressor protein 1
FTH	ferritin heavy chain
GAA	gossypol acetic acid
GCH1	GTP cyclohydrolase 1
GLS	glutaminases
GPX4	glutathione peroxidase 4
GSK3β	glycogen synthase kinase 3 beta
HC	histochrome
HO-1	heme oxygenase 1
I/R	ischemia-reperfusion
IDO	indoleamine 2,3-dioxygenase 1
IRE1	inositol requiring enzyme 1
IRI	ischemia-reperfusion injury
JNK	Jun N-terminal kinase
Lgmn	legumain
LH	polyunsaturated lipid
LIP	labile iron pool
LOO·	lipid peroxyl radical
LOOH	hydroperoxide
LPCAT3	lysophosphatidylcholine acyltransferase 3
LSD1	lysine-specific demethylase 1
MAPK	mitogen-activated protein kinase
MCAO	middle cerebral artery occlusion
MCTR1	maresin conjugate in tissue regeneration 1
MDA	malondialdehyde
MDM2	murine double minute 2
METs	macrophage extracellular traps
MUFA	monounsaturated fatty acid
NADPH	nicotinamide adenine dinucleotide phosphate
NAR	naringenin
NCOA4	nuclear receptor coactivator 4
NOX4	NADPH oxidase 4
NRF2	nuclear factor erythroid 2-related factor 2
NTBI	non-TF-bound iron
OGD/R	oxygen-glucose deprivation/reoxygenation
OH·	hydroxyl radical
OxPCs	oxidized phosphatidylcholines
OXPHOS	oxidative phosphorylation
Panx1	pannexin 1
PCBP	poly(rC)-binding protein
PGE2	prostaglandin E2
PLOO·	phospholipid hydroperoxyl radical
POR	cytochrome P450 oxidoreductase
PTGS2	prostaglandin-endoperoxide synthase 2
PUFAs	polyunsaturated fatty acids
PUM2	pumilio 2
QCT	quercetin
Res	resveratrol
ROS	reactive oxygen species
SIRT1	sirtuin 1
SLC7A11	solute carrier family 7 member 11
SLC25A28	solute carrier family 25 member 28
SLC25A37	solute carrier family 25 member 37
SLC40A1/FPN	solute carrier family 40 member 1
Sp1	special protein 1
TF	transferrin
TFR1	transferrin receptor 1
TLR4	toll like receptor 4
USC	urine-derived stem cells
VDAC1	voltage dependent anion channel 1
